# Impact of Disease Severity and Disease-Modifying Therapies on Myostatin Levels in SMA Patients

**DOI:** 10.3390/ijms25168763

**Published:** 2024-08-12

**Authors:** Laurane Mackels, Virginie Mariot, Laura Buscemi, Laurent Servais, Julie Dumonceaux

**Affiliations:** 1 Adult Neurology Department, Citadelle Hospital, 1 Boulevard Du 12e De Ligne, 4000 Liege, Belgium; 2 MDUK Oxford Neuromuscular Center, Department of Paediatrics, NIHR Oxford Biomedical Research Centre, University of Oxford, Oxford OX3 9DU, UK; laurent.servais@paediatrics.ox.ac.uk; 3 NIHR Great Ormond Street Hospital Biomedical Research Centre and Great Ormond Street Institute of Child Health, University College London, London WC1N 1EH, UK; virginie.mariot@ucl.ac.uk; 4 Neuromuscular Center, Citadelle Hospital, 1 Boulevard Du 12e De Ligne, 4000 Liege, Belgium; laura.buscemi@citadelle.be; 5 Neuromuscular Center, Division of Paediatrics, University Hospital of Liège, University of Liège, Boulevard Du 12e De Ligne, 4000 Liege, Belgium

**Keywords:** spinal muscular atrophy, myostatin, GDF8, follisatin, FSTN, nusinersen, disease-modifying therapies, clinical trials

## Abstract

Clinical trials with treatments inhibiting myostatin pathways to increase muscle mass are currently ongoing in spinal muscular atrophy. Given evidence of potential myostatin pathway downregulation in Spinal Muscular Atrophy (SMA), restoring sufficient myostatin levels using disease-modifying treatments (DMTs) might arguably be necessary prior to considering myostatin inhibitors as an add-on treatment. This retrospective study assessed pre-treatment myostatin and follistatin levels’ correlation with disease severity and explored their alteration by disease-modifying treatment in SMA. We retrospectively collected clinical characteristics, motor scores, and mysotatin and follistatin levels between 2018 and 2020 in 25 Belgian patients with SMA (SMA1 (*n* = 13), SMA2 (*n* = 6), SMA 3 (*n* = 6)) and treated by nusinersen. Data were collected prior to treatment and after 2, 6, 10, 18, and 30 months of treatment. Myostatin levels correlated with patients’ age, weight, SMA type, and motor function before treatment initiation. After treatment, we observed correlations between myostatin levels and some motor function scores (i.e., MFM32, HFMSE, 6MWT), but no major effect of nusinersen on myostatin or follistatin levels over time. In conclusion, further research is needed to determine if DMTs can impact myostatin and follistatin levels in SMA, and how this could potentially influence patient selection for ongoing myostatin inhibitor trials.

## 1. Introduction

Spinal muscular atrophy (SMA) is a recessively inherited neuromuscular condition caused by mutations in the survival motoneuron gene (*SMN1*), resulting in the absence of survival motor neuron protein (SMN) [1]. The absence of SMN protein is partially compensated by an autologous gene, *SMN2*, that is responsible for the production of functional SMN protein, and directly influences SMA disease severity [2,3]. SMA primarily affects motor neurons in both the central and peripheral nervous system, leading to proximal muscle weakness, hypotonia, and muscle atrophy [4,5]. Traditionally, SMA has been divided into five main phenotypes based on age of symptom onset and highest level of motor function achieved [6,7], although these phenotypes tend to evolve with the increasing use of disease-modifying therapies (DMTs) and improved standard of care [8,9,10]. The SMA therapeutic landscape has undergone tremendous progress in the last 10 years, with the development of three DMTs and the implementation of newborn screening programs (NBSs) in several countries [11]. DMTs for SMA differ in their administration route and tissue distribution. Nusinersen, an antisense oligonucleotide, targets the central nervous system through intrathecal delivery [12,13,14]. Onasemnogene abeparvovec (Zolgensma), a gene replacement therapy [15], and risdiplam, a small-molecule splicing modifier [16,17], achieve systemic distribution through intravenous and oral routes, respectively. A large body of evidence has demonstrated a better impact of treatment when initiated within the first days of life, which has paved the way for implementation of NBS [18,19]. While older patients can still benefit from these treatments, the effect is less pronounced [20]. Importantly, even among early-treated patients, treatment benefits can vary, and some patients may still experience varying degrees of motor dysfunction. Beyond muscle function, other issues may persist despite treatment, including cognitive and language impairments [21,22,23]. All these remaining challenges justify ongoing efforts to develop new therapies and add-on therapies to further improve patient outcomes [24,25].

Myostatin, or growth differentiation factor-8 (GDF8), is a member of the transforming growth factor-β (TGFβ) family, that acts as a negative regulator of skeletal muscle bulk and inhibits skeletal muscle growth [26]. Although there is interest in leveraging myostatin inhibition to enhance muscle mass and function in several muscle-wasting diseases, the efficacy of anti-myostatin drugs in clinical trials remains limited [27,28]. Some evidence supports a down-regulation of myostatin pathways in SMA, leading to intrinsically low blood levels of myostatin and high levels of antagonist follistatin (FSTN) [29]. Higher myostatin predicted better anti-myostatin treatment outcomes in animals [30,31], suggesting that restoring sufficient levels of myostatin may be required prior to the inhibition of the myostatin pathways [29]. This information taken together raises relevant questions in the era of ongoing clinicals trials with several anti-myostatin drugs in SMA, as they could impact patient selection: (I) Does myostatin and its antagonist follistatin correlate with disease severity in SMA patients before and after DMTs? (II) Do DMTs influence the levels of myostatin and follistatin in treated patients? (III) Could myostatin and follistatin levels potentially guide patient selection in anti-myostatin therapy? Here, we investigated whether myostatin and follisatin levels correlate with phenotype and motor function prior to, and after, initiation and whether administration of nusinersen in SMA patients impacts myostatin or follistatin levels over time.

## 2. Results

### 2.1. Patients’ Demographics and Clinical Characteristics

We included 13 patients with SMA 1, six patients with SMA 2, and six patients with SMA 3. The median age at treatment initiation was 10.3 years (1 month—59.5 years). *SMN2* copy number was unavailable for two patients; four patients had two copies; 13 had three copies; and six had four copies. At baseline, myostatin and follistatin levels were available for 22 patients (220–3036.4 pg/mL) and 11 patients (834.5–2927.0 pg/mL), respectively. One patient’s follistatin levels were excluded due to aberrant values that could not be verified or explained by any identifiable confounding factor. Median clinical and biological follow-ups were 18.5 months (1–33 months) and 15.5 months (1–29 months), respectively (Supplementary Materials Table S1).

### 2.2. Myostatin and Follistatin Levels at Baseline in Nusinersen-Naïve Patients

#### 2.2.1. Myostatin and Follistatin Levels Per SMA Type and *SMN2* Copy Number

**Figure 1:**
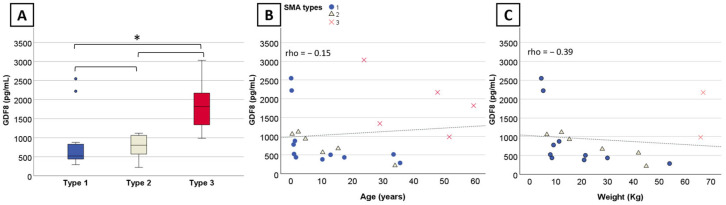
Myostatin levels per SMA type and correlation with age and weight in nusinersen-naïve patients. (**A**) Boxplot displaying myostatin levels for SMA types with outliers indicated by dots. (**B**) Significant correlation between myostatin levels, (**B**) age, and (**C**) weight within SMA1 and SMA2. Significant results are indicated by an asterisk (*) and dots indicate outliers.

We observed a significant difference in myostatin levels across the three SMA types (*n* = 22, η^2^ = 0.729, *p* = 0.03). SMA type 3 exhibited significantly higher (*p* = 0.03) myostatin levels (Md = 1818.4 pg/mL) compared to type 1 (Md = 517.8 pg/mL). The difference between type 3 and type 2 (Md = 802.6 pg/mL) was not significant (*p* = 0.19), although the small sample size may underestimate the difference (Figure 1A). There was no significant difference in myostatin levels between SMA type 1 and type 2 (*p* = 1). Despite significant overall group differences in myostatin levels across the different *SMN2* copy number (*n* = 20, η^2^ = 0.76, *p* = 0.04), pairwise comparison remained non-significant. No significant difference was observed in the follistatin levels across SMA type (*n* = 11, *p* = 0.52) and *SMN2* copy number (*n* = 11, *p* = 0.37).

#### 2.2.2. Correlation between Myostatin and Follistatin Levels and Baseline Characteristics

**Figure 2:**
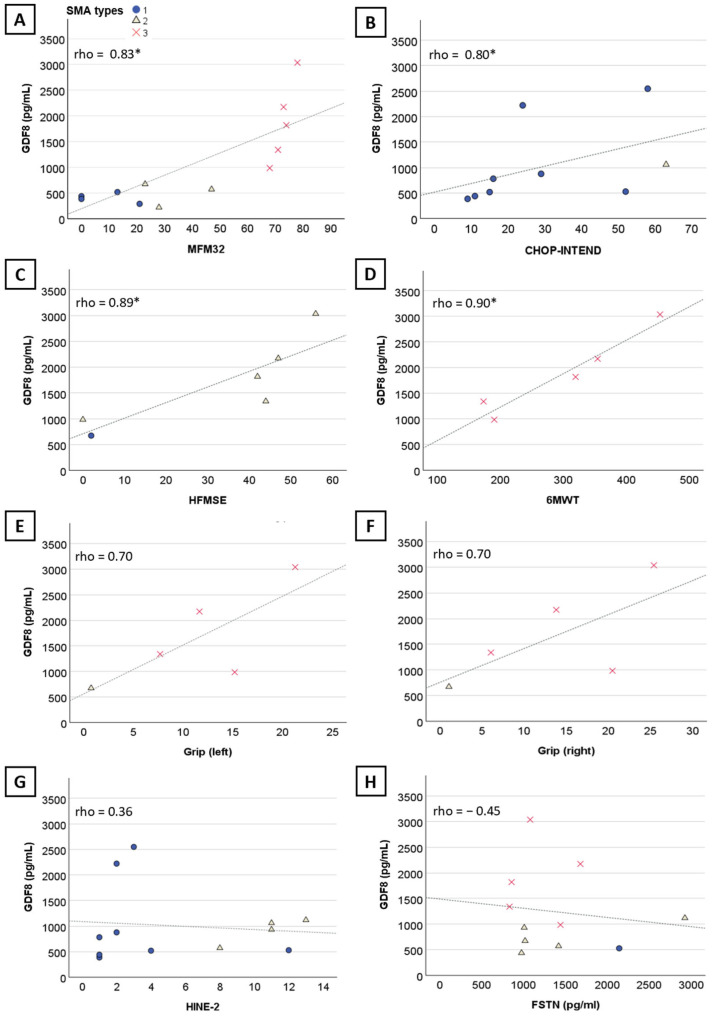
Correlation of myostatin levels with motor scores and follistatin blood levels in nusinersen-naïve patients. Graphs showing the significant correlation of myostatin with (**A**) MFM32, (**B**) CHOP-INTEND, (**C**) HFMSE, (**D**) 6MWT. No significant correlation was observed between myostatin and left grip score (**E**), right grip score (**F**), HINE-2 (**G**), and follistatin (**H**). Significant results are indicated by an asterisk (*).

There was no significant correlation between myostatin levels and age (*n* = 22, rho = −0.15, 95%CI [−0.58; 0.35], *p =* 0.5) (Figure 1B) or weight (*n* = 18, rho = −0.39, 95%CI [−0.85; 0.22], *p =* 0.11) (Figure 1C) when considering the three SMA types altogether. However, myostatin levels negatively correlated with age and weight at baseline in SMA type 1 (*n* = 11, rho = −0.84, 95%CI [−1; −0.38], *p* = 0.001 and *n* = 10, rho = −0.81, 95%CI [−1; −0.30], *p* = 0.005, respectively) and type 2 (*n* = 6, rho = −0.89, 95%CI [−1; −0.34], *p* = 0.02 and *n* = 6, rho = −0.94, 95%CI [−1; −0.51], *p* = 0.05, respectively). Myostatin correlated with several motor scores (32-item Motor Function Measure (MFM32) (*n* = 12, rho = 0.83, 95%CI [0.31; 0.99], *p* < 0.001) (Figure 2A), Children’s Hospital of Philadelphia Infant Test of Neuromuscular-Disorders (CHOP-INTEND) (*n* = 9, rho = 0.80, 95%CI [0.13; 1], *p* = 0.01) (Figure 2B), Hammersmith Functional Motor Scale Expanded (HFMSE) (*n* = 6, rho = 0.89, 95%CI [0.20; 1], *p* = 0.02) (Figure 2C), and Six-Minute Walk Test (6MWT) (*n* = 5, rho = 0.90, 95%CI [0.11; 1], *p* = 0.04) (Figure 2D). No significant correlation was observed with the Hammersmith Infant Neurological Examination (HINE-2) (*n* = 12, rho = 0.36, 95%CI [−0.35; 0.84) (Figure 2G), although two outliers treated within two months of life might have skewed the results. No correlation was found with left (*n* = 5, rho = 0.7, 95%CI [−0.875; 1 pg/mL]) (Figure 2E) and right grip score (*n* = 5, rho = 0.7, 95%CI [−0.936; 1) (Figure 2F), and follistatin levels (*n* = 11, rho = −0.045, 95%CI [−0.628; 0.653]) (Figure 2H).

### 2.3. Change in Myostatin and Follistatin Levels over Time in Treated Patients

**Figure 3:**
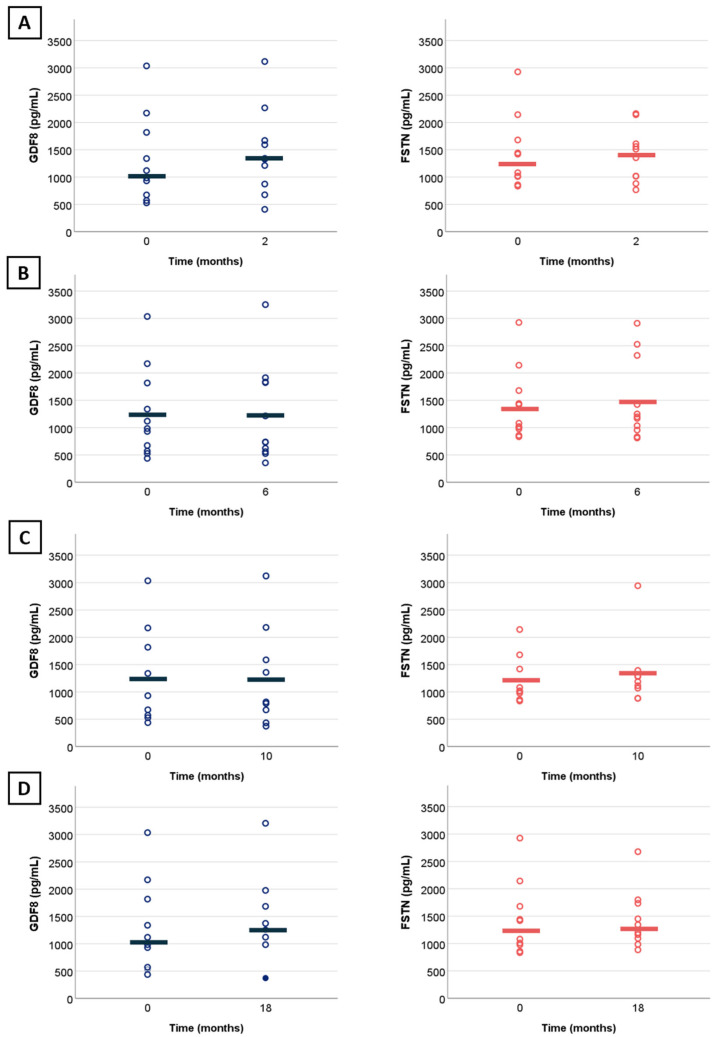
Change in myostatin and follistatin levels from baseline in treated patients. (**A**) Change in myostatin (blue) and follistatin (red) levels over a period of 2 months with treatment by nusinersen (N = 10); (**B**) over a period of 6 months with treatment by nusinersen (N = 11); (**C**) over a period of 10 months with treatment by nusinersen (N = 9); (**D**) over a period of 18 months with treatment by nusinersen (N = 10). Full dots indicate overlapping points. Horizontal lines illustrate mean values.

**Figure 4:**
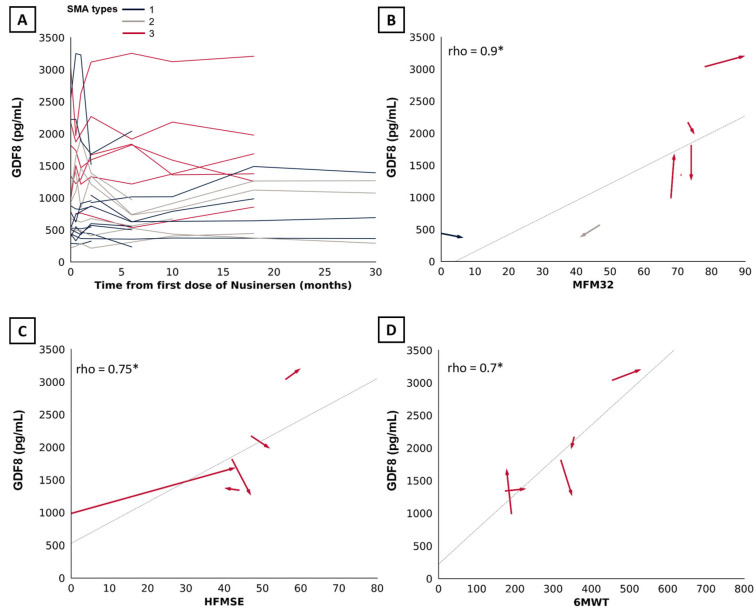
Association between myostatin and motor scores over time. (**A**) Line graph displaying the trend in myostatin levels following up to 30 months of treatment with nusinersen (n = 25). (**B**) Arrow graph showing the association between myostatin and MFM32, (**C**) HFMSE, and (**D**) 6MWT from baseline to 18 months of treatment with nusinersen. Arrow indicates temporality. SMA types 1, 2, and 3 are indicated in blue, beige, and red, respectively. Significant results are indicated by an asterisk (*).

SMA types were analyzed altogether given the small sample size. We observed no significant changes in myostatin (*n* = 10, r = 0.11, estimate of 61.2 pg/mL, 95%CI: [−194.2; 316.8 pg/mL], *p* = 0.77) and follistatin levels (*n* = 10, r = 0.11, estimate of 92.5 pg/mL, 95%CI: [−469.85; 437.15 pg/mL], *p* = 0.77) at 18 months of treatment (Figure 3D). Change were non-significant at additional time points of 2 months (*n* = 10, r = 0.56, estimate of 151.6 pg/mL, 95%CI: [−33.4; 305.8 pg/mL] and r = 0.16, estimate of 20 pg/mL, 95%CI [−544.6; 360.5 pg/mL]) (Figure 3A), 6 months (*n* = 11, r = −0.05, estimate of −16.2 pg/mL, 95%CI: [−185.2; 192.0 pg/mL] (Figure 3B) and r = −0.08, estimate of −50.25 pg/mL, 95%CI [−243.5; 649.2 pg/mL]), and 10 months (*n* = 9, r = −0.14, estimate of −25.2 pg/mL, 95%CI [−125.6; 98.2 pg/mL] and r = 0.33, estimate of 70 pg/mL, 95%CI [−178; 506 pg/mL]) (Figure 3C). Statistical analysis at 30 months was not possible due to the small sample size (*n* = 4). Aligning with these findings, the line graph confirms no specific trend in myostatin level changes following up to 30 months of nusinersen. (Figure 4A). We observed a correlation at 18 months of treatment between myostatin and MFM32 (*n* = 14, rho = 0.9, 95%CI [0.62; 0.99], *p* = <0.001) (Figure 3B), HFMSE (*n* = 5, rho = 0.75, 95%CI [0.98; 0.99], *p* = 0.01) (Figure 4C), and 6MWT (*n* = 5, rho = 0.7, 95%CI [0.04–1], *p* = 0.01) (Figure 4D). There was no correlation with follistatin, nor between follistatin and motor scores (Supplementary Materials Table S2).

## 3. Discussion

In a small patient sample, we observed an inverse correlation between myostatin levels, age, and weight at baseline in both SMA1 and SMA2 patients. Additionally, our findings support prior research, showing correlations between myostatin levels and disease severity, as evidenced by lower myostatin levels in SMA1 patients, and correlation with several motor scores [32,33].

Myostatin and follistatin are growth and differentiation factors belonging to the transforming growth factor-beta (TGF-beta) superfamily. Myostatin RNA is specifically expressed in developing and mature muscle tissues in mice [34]. During embryogenesis, expression is restricted to the myotome, with more widespread muscular expression observed in adult animals [34]. Myostatin, like other TGF-β members, is initially expressed as a precursor protein that undergoes cleavage into an N-terminal propeptide and a disulfide-linked C-terminal dimer, which is the biologically active molecule. The circulating form of myostatin in the bloodstream consists of a latent complex of the myostatin C-terminal dimer and other proteins, including the inhibitory myostatin propeptide [35,36]. Cleavage of the propeptide by a metalloproteinase activates latent myostatin, allowing it to bind to its receptors, ActRIIA and ActRIIB [37,38]. Follistatin acts as a myostatin antagonist through direct protein–protein interaction that sequesters myostatin, preventing its binding to the activin type IIB receptor [39,40]. Myostatin’s role as a negative regulator of skeletal muscle mass was initially highlighted in *GDF8*-null mice, which displayed a significant increase in weight and muscle mass due to increased fiber size and fiber number [34]. This increase in muscle bulk was then observed as a result of *MSTN* mutations across different animal species [41,42,43] and humans [44]. Concomitantly, agents designed to block myostatin production in adult mouse models [45] confirmed the increase in muscle mass following myostatin inhibition, including in disease models of Becker (BMD) and Duchenne muscular dystrophy (DMD) [31,46,47], dysferlinopathy [48], limb-girdle muscular dystrophy type 2A (LGMD2A) [49], calpainopathy [50], and SMA [29,51,52,53,54]. Altogether, these results have generated enthusiasm regarding the potential of inhibiting myostatin and its pathway to increase muscle mass and motor function in muscle-wasting conditions.

Three drugs targeting the myostatin signaling pathway are currently being evaluated in clinical trials (Table 1). SRK-015 (Scholar Rock^®,^, Cambridge, MS, USA) is a monoclonal antibody that selectively inhibits pro- and latent myostatin. It has completed phase 1 (NCT02644777) and phase 2 (TOPAZ, NCT03921528) trials [55,56], as well as a 12-month randomized, controlled phase 3 trial (RCT) in patients with type 2 and type 3 SMA, undergoing treatment by nusinersen or risdiplam. An open-label extension study is currently ongoing for patients who completed the two aforementioned trials (ONYX, NCT05626855). The TOPAZ trial (NCT03921528) demonstrated sustained improvement with apitegromab and nusinersen in non-ambulatory patients, using HFMSE, Revised Upper Limb Module (RULM), and WHO motor development milestones as functional outcomes [57]. Taldefgrobep alfa (Biohaven^®^, New Haven, CT, USA) is a humanized recombinant protein designed to neutralize free myostatin and block the activin IIb receptor. This dual action inhibits the signaling of both myostatin and activin A [58]. Preclinical data and a well-established safety profile exist from studies in patients with neuromuscular diseases [59], including DMD (NCT03039686, NCT02515669) [58]. A randomized, double-blind, placebo-controlled phase 3 trial (RESILIENCE, NCT05337553) is currently underway in both ambulatory and non-ambulatory patients. Notably, unlike SRK-015 trials, RESILIENCE allows for the inclusion of patients with a history of onasemnogene abeparvovec-xioi treatment [60]. RO7204239 (Roche^®^, Basel, Switzerland), a monoclonal anti-myostatin antibody designed to eliminate myostatin from plasma and tissues, is currently being investigated in a phase 2 and phase 3 RCT (MANATEE, NCT05115110). This two-part study is evaluating the safety and efficacy of the antibody when used in combination with risdiplam in ambulatory and non-ambulatory patients [61].

Excluding a few small open-label studies and RCTs [62,63,64], therapies targeting the myostatin pathway have yielded inconclusive results in DMD [27,65] and other adult neuromuscular diseases [66,67,68]. One plausible explanation is the downregulation of the pathway itself in muscle-wasting diseases, limiting the availability of therapeutic targets [29]. Evidence for this comes from the reduced myostatin pathway mRNA expression observed in skeletal muscles of neuromuscular patients with severe muscle loss, such as those with SMA and DMD [29]. Higher myostatin predicted better anti-myostatin treatment outcomes in animals [28,29,30,31], suggesting that restoring sufficient levels of myostatin may be required prior to the inhibition of the myostatin pathways [29]. Moreover, SMA animal models suggest greater benefit from myostatin-targeting drugs combined with SMN-restoring therapies [51,52,69]. Several key questions remain regarding myostatin and its role in SMA treatment. Firstly, there is a need to determine whether myostatin levels accurately reflect the severity of the phenotype and the patient’s response to treatment. Secondly, only a few studies have investigated the impact of DMTs on myostatin levels so far. Finally, it is still unclear whether myostatin levels could serve as a biomarker for selecting patients who might respond best to anti-myostatin therapies. If achieving minimum blood levels of myostatin proves to be a prerequisite for the effectiveness of anti-myostatin therapies, then identifying which patients are likely to fall into this category becomes crucial. Thus, analyzing blood levels of myostatin in SMA patients across different SMA subtypes, *SMN2* copies, and during DMTs could be highly informative for future trials.

Our data support previous findings, which demonstrated an inverted correlation between myostatin levels and disease severity in SMA [32,33] and other neuromuscular disorders [29,70,71]. This aligns with the observation of lower myostatin levels in neuromuscular patients compared to healthy controls [72]. Our findings suggest a trend of decreasing myostatin levels with age in SMA types I and II, but not in SMA type III, which has a milder phenotype. Similarly, a significant decrease in myostatin levels with age was shown in DMD patients, while the decrease was significant but less pronounced in BMD patients [72]. However, this relationship remains unclear due to inconsistent results across studies [33,70]. The correlation between myostatin levels and baseline age in our cohort might not reflect a true age-related decline. The lack of a clear trend in myostatin levels over time within individual patients could indicate either no correlation with age or a stabilization of myostatin levels following treatment. No correlation of follistatin with phenotype, type, or age and no trends in blood levels over time were observed, consistent with recent published studies in SMA [33].

Myostatin has been suggested as a potential biomarker for monitoring disease progression in several neuromuscular diseases, including SMA [30,32,33,73,74]. Our findings suggest that myostatin levels correlate with motor function, before and after treatment. However, the small sample size and potential confounding factors need to be addressed in future research. We did not observe significant changes in serum levels of follistatin and myostatin over time in patients treated with nusinersen, contrasting with a recent study reporting ongoing decreases in myostatin levels over time [33]. Limited nusinersen efficacy in SMA, small sample size, and intrathecal delivery’s potential lack of effect on systemic myostatin levels are possible explanations. Future studies with systemic treatments in SMA patients might offer valuable insights.

Several limitations of our study must be acknowledged. The retrospective design and small sample size limited statistical power. Given the mixed-age cohort and inclusion of three SMA subtypes, we may have missed subtle or transient changes, especially within specific SMA subgroups. Data gaps due to disruption in follow-up and unavailable samples during COVID-19 further reduced statistical power. Short follow-up periods in some patients might have missed slower changes. Additionally, we did not control for age at treatment, weight, or body lean mass, and the lack of robust statistical models to adjust for confounding variables limits our conclusions.

Nevertheless, understanding myostatin pathways in treated SMA patients remains crucial in the era of newborn screening and ongoing clinical trials. Future research should explore the correlation of myostatin with clinical metrics such as standardized motor scores reflecting patient phenotypes. Criteria defining therapeutic response should be established upfront and tailored to each SMA subgroup or SMA copy number, as the boundaries between classical SMA types are blurring with the implementation of DMTs and newborn screening. These criteria should be standardized across studies to minimize variability. Statistical corrections for multiple comparisons [75], and robust statistical models for repeated measures, such as linear mixed-effects models [76] adjusted for age, lean mass, and weight, should be employed. Longer follow-up periods will help capture slower fluctuations, and we believe a 12-month period of stable nusinersen dosage might be considered for phenotype assessment, based on previous studies evaluating clinical response [77].

## 4. Methods

### Patient Characteristics and Study Protocol

In this retrospective monocentric study, we gathered clinical data and serum myostatin and follistatin levels from 25 Belgian SMA patients treated with intrathecal nusinersen at a standard dosing regimen in Citadelle Hospital, Liège, Belgium, between 2018 and 2020. The study received approval from the institutional Ethical Committee of the Citadelle Hospital (reference: JL/rc/2105). Data were collected at multiple time points (baseline, 14 days, 1, 2, 6, 10, 18, and 30 months) and values were averaged within a ±25% interval around each specific time point. Clinical data collected from medical records included gender, SMA type, number of SMN2 copies, age at treatment initiation, weight, BMI, and number of *SMN2* copies. When available, motor scores were collected for CHOP-INTEND, HINE-2, HFSME, MFM32, Six-Minute Walk Test (6MWT), and grip strength test. Peripheral venous samples were collected as part of the standard of care for nusinersen treatment, using serum separator tubes (10 mL). Patients’ parents agreed to have leftover fluids used for future research. Myostatin and follistatin levels were assessed at UCL Great Ormond Street Institute of Child Health. After 30 min at room temperature, tubes were centrifuged at 2000 rpm for 10 min at 4 °C. The collected serum (5 mL) was aliquoted and stored at −80 °C.

Myostatin and follistatin concentrations in the sera were measured using an ELISA kit following the manufacturer’s instructions and previous methodology [29]. Optical density was measured with a microplate reader (Infinite 200 Pro, Tecan Group Ltd., Männedorf, Switzerland).

Given the small sample size, we performed non-parametric tests (*p* < 0.05). Spearman correlations and bootstrapping for a 95% confidence interval (95%CI) were used for the correlation between myostatin and follistatin levels and clinical parameters. Myostatin and follistatin levels across SMA types and *SMN2* copy numbers were compared using the Kruskal–Wallis test with Dunn’s post hoc tests for pairwise differences. Bonferroni correction was adjusted for multiple comparisons, and the effect size was assessed with eta squared (η^2^). We reported the Wilcoxon signed-rank test along with Wilcoxon effect size (r) [78], Hodges–Lehmann estimator, and 95%CI for changes in myostatin and follistatin levels over time (2, 6, 10, and 18 months).

## Figures and Tables

**Table 1:** Anti-myostatin therapies in spinal muscular atrophy. HFMSE: Hammersmith Functional Motor Scale Expanded, MFM32: 32-item Motor Function Measure, N/A: Not Applicable, RHS: Revised Hammersmith Scale, Y: years old.

	Apitegromab	Taldefgrobep Alfa	RO7204239
Manufacturer	Scholar Rock	Biohaven	Roche
Mechanism	Fully human monoclonal antibody targeting inactive precursor forms of myostatin and pro- and latent myostatin	Fully human anti-myostatin antibody, targeting the C-terminal of mature myostatin and the ActRIIB–myostatin complex	Recycling and sweeping humanized antibody targeting latent myostatin
Clinical Trail	Phase II (TOPAZ, NCT03921528) Phase III (SAPPHIRE, NCT05156320) Open label access (ONYX, NCT05626855)	Phase III (RESILIENT, NCT05337553)	Phase II (MANATEE; NCT05115110) Phase III (MANATEE; NCT05115110)
Delivery Route	IV	SC	SC
Dosing Frequency	1×/4 weeks	1×/week	1×/4 weeks
Age range	TOPAZ: 2–21 y SAPPHIRE: ≥2 y ONYX: ≥2 y	4–21 y	Part 1: 2–10 y (ambulant), 5–10 y (non-ambulant) Part 2: 2–25 y
SMA types	Type II and type III	Any	All
Ambulatory Status	TOPAZ: Ambulatory and non-ambulatory SAPPHIRE: Non-ambulatory ONYX: Non-ambulatory	Ambulant or non-ambulant	Part 1: Ambulant and non-ambulant Part 2: Ambulant
Concomitant treatment	Nusinersen or risdiplam	Spinraza or Evrysdi and/or history of Zolgensma	Evrysdi and/or history of treatment with Zolgensma
Motor score used as primary outcomes	TOPAZ: RHS, HFMSE SAPPHIRE: HFMSE ONYX: N/A	MFM32	RHS
Completion date	TOPAZ: February 2024 SAPPHIRE: December 2024 ONYX: 27 January	25 January	26 June

## Data Availability

The data presented in this study are available on request from the corresponding authors due to the General Data Protection Regulation.

## Supplementary Materials

The following supporting information can be downloaded at https://www.mdpi.com/article/10.3390/ijms25168763/s1.

## Abbreviations

6MWT	Six-Minute Walk Test
BMD	Becker muscular dystrophy
CHOP-INTEND	Children’s Hospital of Philadelphia Infant-Test-of-Neuromuscular-Disorders
DMD	Duchenne muscular dystrophy
DMTs	Disease-modifying therapies
FSTN	Follistatin
GDF8	Growth differentiation factor-8
HFMSE	Hammersmith Functional Motor Scale Expanded
HINE-2	Hammersmith Infant Neurological Examination 2
LGMD2A	Limb-girdle muscular dystrophy type 2A
MFM32	32-item Motor Function Measure
NBS	Newborn screening
RCT	Randomized control trial
RHS	Revised Hammersmith Scale
RULM	Revised Upper Limb Module
SMA	Spinal muscular atrophy
SMN	Survival motor neuron
TGFβ	Transforming growth factor-β
