# Supplementary material for: Impact of Disease Severity and Disease-Modifying Therapies on Myostatin Levels in SMA Patients
Source: Int J Mol Sci. 2024 Aug 12;25(16):8763. doi: 10.3390/ijms25168763 (PMC11354404; doi:10.3390/ijms25168763)
Supplement: Supplementary file 1 [file ijms-25-08763-s001.zip › Supplementary Materials Table S1.pptx]

## Slide 1
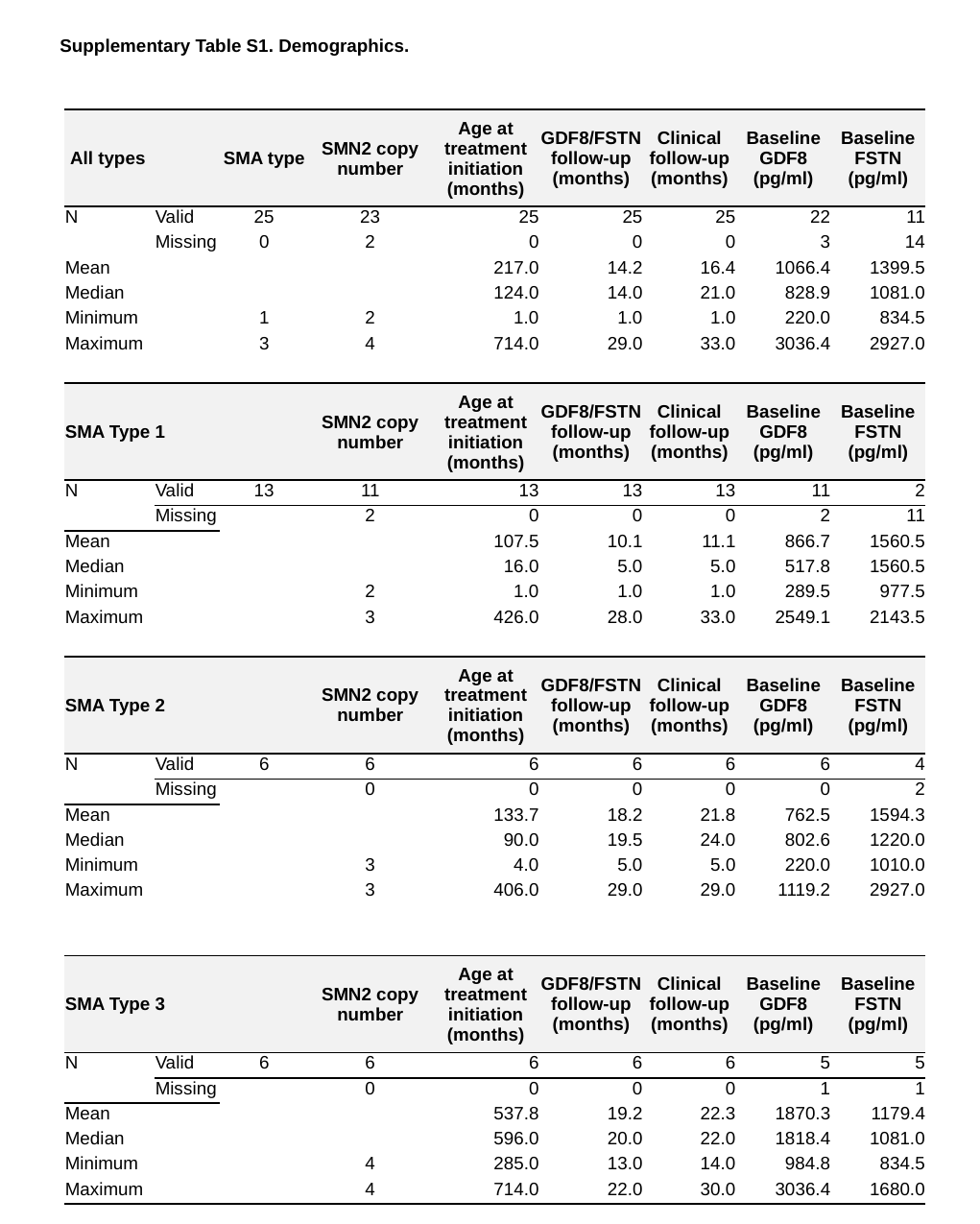

Supplementary Table S1. Demographics.
| All types | | SMA type | SMN2 copy number | Age at treatment initiation (months) | GDF8/FSTN follow-up (months) | Clinical follow-up (months) | Baseline GDF8 (pg/ml) | Baseline FSTN (pg/ml) |
| --- | --- | --- | --- | --- | --- | --- | --- | --- |
| N | Valid | 25 | 23 | 25 | 25 | 25 | 22 | 11 |
| | Missing | 0 | 2 | 0 | 0 | 0 | 3 | 14 |
| Mean | | | | 217.0 | 14.2 | 16.4 | 1066.4 | 1399.5 |
| Median | | | | 124.0 | 14.0 | 21.0 | 828.9 | 1081.0 |
| Minimum | | 1 | 2 | 1.0 | 1.0 | 1.0 | 220.0 | 834.5 |
| Maximum | | 3 | 4 | 714.0 | 29.0 | 33.0 | 3036.4 | 2927.0 |
| | | | | | | | | |
| SMA Type 1 | | | SMN2 copy number | Age at treatment initiation (months) | GDF8/FSTN follow-up (months) | Clinical follow-up (months) | Baseline GDF8 (pg/ml) | Baseline FSTN (pg/ml) |
| N | Valid | 13 | 11 | 13 | 13 | 13 | 11 | 2 |
| | Missing | | 2 | 0 | 0 | 0 | 2 | 11 |
| Mean | | | | 107.5 | 10.1 | 11.1 | 866.7 | 1560.5 |
| Median | | | | 16.0 | 5.0 | 5.0 | 517.8 | 1560.5 |
| Minimum | | | 2 | 1.0 | 1.0 | 1.0 | 289.5 | 977.5 |
| Maximum | | | 3 | 426.0 | 28.0 | 33.0 | 2549.1 | 2143.5 |
| | | | | | | | | |
| SMA Type 2 | | | SMN2 copy number | Age at treatment initiation (months) | GDF8/FSTN follow-up (months) | Clinical follow-up (months) | Baseline GDF8 (pg/ml) | Baseline FSTN (pg/ml) |
| N | Valid | 6 | 6 | 6 | 6 | 6 | 6 | 4 |
| | Missing | | 0 | 0 | 0 | 0 | 0 | 2 |
| Mean | | | | 133.7 | 18.2 | 21.8 | 762.5 | 1594.3 |
| Median | | | | 90.0 | 19.5 | 24.0 | 802.6 | 1220.0 |
| Minimum | | | 3 | 4.0 | 5.0 | 5.0 | 220.0 | 1010.0 |
| Maximum | | | 3 | 406.0 | 29.0 | 29.0 | 1119.2 | 2927.0 |
| | | | | | | | | |
| | | | | | | | | |
| SMA Type 3 | | | SMN2 copy number | Age at treatment initiation (months) | GDF8/FSTN follow-up (months) | Clinical follow-up (months) | Baseline GDF8 (pg/ml) | Baseline FSTN (pg/ml) |
| N | Valid | 6 | 6 | 6 | 6 | 6 | 5 | 5 |
| | Missing | | 0 | 0 | 0 | 0 | 1 | 1 |
| Mean | | | | 537.8 | 19.2 | 22.3 | 1870.3 | 1179.4 |
| Median | | | | 596.0 | 20.0 | 22.0 | 1818.4 | 1081.0 |
| Minimum | | | 4 | 285.0 | 13.0 | 14.0 | 984.8 | 834.5 |
| Maximum | | | 4 | 714.0 | 22.0 | 30.0 | 3036.4 | 1680.0 |
