# Supplementary material for: Impact of Disease Severity and Disease-Modifying Therapies on Myostatin Levels in SMA Patients
Source: Int J Mol Sci. 2024 Aug 12;25(16):8763. doi: 10.3390/ijms25168763 (PMC11354404; doi:10.3390/ijms25168763)
Supplement: Supplementary file 1 [file ijms-25-08763-s001.zip › Supplementary Materials Table S2.pptx]

## Slide 1
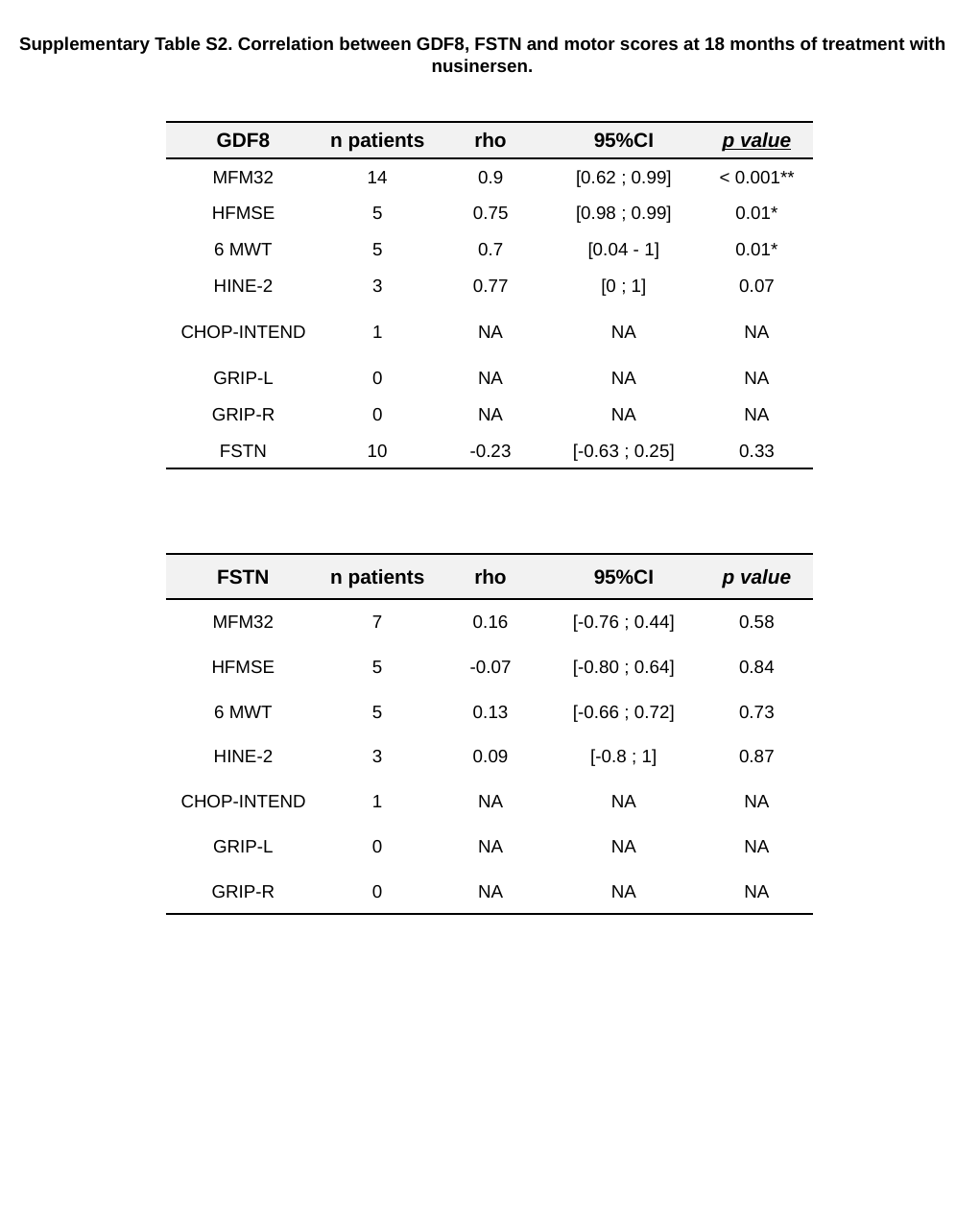

Supplementary Table S2. Correlation between GDF8, FSTN and motor scores at 18 months of treatment with nusinersen.
| GDF8 | n patients | rho | 95%CI | p value |
| --- | --- | --- | --- | --- |
| MFM32 | 14 | 0.9 | [0.62 ; 0.99] | < 0.001\*\* |
| HFMSE | 5 | 0.75 | [0.98 ; 0.99] | 0.01\* |
| 6 MWT | 5 | 0.7 | [0.04 - 1] | 0.01\* |
| HINE-2 | 3 | 0.77 | [0 ; 1] | 0.07 |
| CHOP-INTEND | 1 | NA | NA | NA |
| GRIP-L | 0 | NA | NA | NA |
| GRIP-R | 0 | NA | NA | NA |
| FSTN | 10 | -0.23 | [-0.63 ; 0.25] | 0.33 |
| FSTN | n patients | rho | 95%CI | p value |
| --- | --- | --- | --- | --- |
| MFM32 | 7 | 0.16 | [-0.76 ; 0.44] | 0.58 |
| HFMSE | 5 | -0.07 | [-0.80 ; 0.64] | 0.84 |
| 6 MWT | 5 | 0.13 | [-0.66 ; 0.72] | 0.73 |
| HINE-2 | 3 | 0.09 | [-0.8 ; 1] | 0.87 |
| CHOP-INTEND | 1 | NA | NA | NA |
| GRIP-L | 0 | NA | NA | NA |
| GRIP-R | 0 | NA | NA | NA |
